# Combination Treatment with PPAR*γ* Ligand and Its Specific Inhibitor GW9662 Downregulates BIS and 14-3-3 Gamma, Inhibiting Stem-Like Properties in Glioblastoma Cells

**DOI:** 10.1155/2017/5832824

**Published:** 2017-05-31

**Authors:** Chang-Nim Im

**Affiliations:** Department of Psychiatry, Seoul St. Mary's Hospital, The Catholic University of Korea, Seoul 06591, Republic of Korea

## Abstract

PPAR*γ* is a nuclear receptor that regulates differentiation and proliferation and is highly expressed in many cancer cells. Its synthetic ligands, such as rosiglitazone and ciglitazone, and its inhibitor GW9662, were shown to induce cellular differentiation, inhibit proliferation, and lead to apoptosis. Glioblastoma is a common brain tumor with poor survival prospects. Recently, glioblastoma stem cells (GSCs) have been examined as a potential target for anticancer therapy; however, little is known about the combined effect of various agents on GSCs. In this study, we found that cotreatment with PPAR*γ* ligands and GW9662 inhibited stem-like properties in GSC-like spheres, which significantly express SOX2. In addition, this treatment decreased the activation of STAT3 and AKT and decreased the amounts of 14-3-3 gamma and BIS proteins. Moreover, combined administration of small-interfering RNA (siRNA) transfection with PPAR*γ* ligands induced downregulation of SOX2 and MMP2 activity together with inhibition of sphere-forming activity regardless of poly(ADP-ribose) polymerase (PARP) cleavage. Taken together, our findings suggest that a combination therapy using PPAR*γ* ligands and its inhibitor could be a potential therapeutic strategy targeting GSCs.

## 1. Introduction

Glioblastoma multiform (GBM, also known as glioblastoma) is a common and lethal malignant brain tumor [1]. Glioblastoma stem cells (GSCs) are at the root of tumor recurrence and are regarded as a potential target for anticancer therapy [2, 3].

PPAR*γ* belongs to a nuclear receptor family, along with PPAR*α* and *β*/d, and is highly expressed not only in adipocytes during differentiation [4] but also in various cancer cells [5]. PPAR*γ* ligands such as thiazolidinedione and rosiglitazone induce differentiation and apoptosis in various human glioblastoma cells [6–11]. For instance, Kato et al. showed that 95% of glioma tissue expressed PPAR*γ* mRNA and that a PPAR*γ* ligand, troglitazone, inhibited growth in both SK-MG-1 and NB-1 cell lines [5]. Morosetti et al. reported that human glioblastoma cell lines, such as A172 and U87-MG, also express high levels of PPAR*γ*, which rosiglitazone inhibited proliferation of those cell lines by G2/M arrest and apoptosis, and that the growth inhibitory effect was partially reversed by the PPAR*γ* antagonist GW9662 [9], suggesting that it may work through PPAR*γ*-dependent and PPAR*γ*-independent pathways. PPAR*γ* antagonists, such as GW9662, enhance PPAR*γ* ligand-induced apoptosis [6, 12]. This suggests that both PPAR*γ* ligands [13–16] and GW9662 may be potential agents for glioblastoma therapy specifically targeting GSCs [17, 18].

14-3-3 is an adaptor protein that binds a variety of proteins via a p-Ser/Thr-containing motif. Seven 14-3-3 isoforms (beta, epsilon, zeta, eta, theta, gamma, and sigma) have been identified in mammalian cells. These isoforms are broadly and differentially expressed in almost all tissues and in brain tumors, such as glioblastoma and astrocytoma [19–21]. 14-3-3 beta and sigma are well described, suggesting that these isoforms regulate proliferation in astrocytoma and stem cells, respectively [22, 23]. Jin et al. demonstrated that 14-3-3 gamma interacts with diverse proteins and that this interaction is strengthened by AKT [24], suggesting that AKT may act upstream of 14-3-3 and that they are connected to cancer progression [25]. The antiapoptotic actions of ligand-activated PPAR*γ* are mediated through enhanced binding of PPAR to the promoter of 14-3-3 epsilon and upregulation of 14-3-3 epsilon expression, suggesting that the PPAR to 14-3-3 transcriptional axis plays an important role protecting cell and tissue integrity and may be a possible target for drug discovery [26, 27].

Bcl2-interacting cell death suppressor (BIS) [28], also known as BAG3 [29], has antiapoptotic functions and controls cellular protein quality [30, 31], and it is overexpressed in human glioblastoma tissue [32]. Our recent report demonstrated that BIS is linked to glioblastoma stemness by stabilization of the signal transducer and activator of transcription 3 (STAT3) [33]. These findings provide reliable evidence that BIS is a potential target for therapy. Although PPAR*γ* ligands and/or its antagonists induce apoptosis in cancer cells, including glioblastoma cells [9, 10, 12], the link between 14-3-3 gamma and BIS in GSC-like spheres is not well defined.

Previously, we established a GSC-like sphere culture system in which SOX2 was expressed at significant levels [34] and hypothesized that PPAR*γ* ligands may affect cancer stemness and induce apoptosis in GBM [35]. In this study, we describe the effect of a combination treatment with PPAR*γ* ligands and its inhibitor GW9662 on spheres of glioblastoma cells through downregulation of BIS and 14-3-3 gamma levels, as well as inhibition of SOX2, MMP2 activity, and sphere-forming activity without enhancing the levels of cleaved poly(ADP-ribose) polymerase (PARP).

## 2. Materials and Methods

### 2.1. Cell Culture

A172 and U87-MG (U87) human glioblastoma cells were purchased from American Type Culture Collection (ATCC, Manassas, VA, USA) and maintained in DMEM (HyClone, Logan, UT, USA) contained with 10% heat-inactivated fetal bovine serum (FBS), 100 units/mL penicillin, and 100 mg/mL streptomycin at 37°C in 5% CO_2_ atmosphere. For sphere culture, cells (1 × 10^5^ cells per well) were cultured on ultralow attachment 6-well plate (Corning, Tewksbury, MA) for 72 hours in serum-free glioblastoma sphere medium containing epidermal growth factor (EGF, 20 ng/mL, R&D Systems, Minneapolis, MN) and basic fibroblast growth factor (bFGF, 20 ng/mL, R&D Systems). For morphological examination, spheres per field were counted and pictures were taken under the inverted microscope.

### 2.2. Sphere-Formation Assay

Spheres cultivated in serum-free glioblastoma sphere medium containing EGF and bFGF were attached to standard culture plates in media containing 5% FBS stained with crystal violet solution (Sigma-Aldrich, St. Louis, MO, USA). For morphological examination, spheres per field were counted and pictures were taken under the inverted microscope.

### 2.3. Cell Viability Assay

Cell proliferation was assessed as a function of metabolic activity using an EZ-Cytox Cell Viability Assay Kit (ItsBio, Seoul, Korea). The assay is based on reduction of tetrazolium chloride to the water-soluble formazan by succinate-tetrazolium reductase, which forms part of the mitochondrial respiratory chain. After treatment with 20 *μ*L per well, cells were incubated for 2 hours at 37°C in a 5% CO_2_ atmosphere. Absorbance was measured on a microplate reader (Bio-Rad 680; Bio-Rad, Hercules, CA, USA) at 450 nm. After subtraction of the background, the viability was determined as the ratio relative to the control and reported as the mean±standard error (SE).

### 2.4. Western Blot

Cells were lysed with RIPA buffer (150 mM NaCl, 1% NP-40, 0.5% sodium deoxycholate, 0.1% SDS, 50 mM Tris–HCl pH 8.0) with protease inhibitor (Roche Diagnostics, Mannheim, Germany) on ice for 30 m. Equal amounts of protein were separated on 10% sodium dodecyl sulphate polyacrylamide gel electrophoresis (SDS-PAGE) and transferred to nitrocellulose membranes (GE Healthcare Life Sciences, Buckinghamshire, UK). The membranes were incubated for 1 h with 5% dry skim milk in TBST (20 mM Tris, 137 mM NaCl, and 0.1% Tween 20) buffer and then incubated with antibodies against BIS [28], p-STAT3 (Y705), p-AKT (S473), and PPAR*γ* (Cell Signaling, Danvers, Massachusetts, USA), SOX2 (Santa Cruz Biotechnology), cleaved PARP (Abcam, Cambridge, UK), or beta-actin (Sigma-Aldrich, St. Louis, MO, USA). After incubation with horseradish peroxidase-conjugated anti-mouse or anti-rabbit IgG (1 : 5,000; Santa Cruz Biotechnology), the immunoreactive bands were visualized by an enhanced chemiluminescence substrate (ThermoFisher Scientific). Quantification of the intensities of each band was carried out using ImageJ software (National Institutes of Health, Bethesda, MD, USA).

### 2.5. Small-Interfering RNA (siRNA) Transfection

Knockdown was performed by transfection of specific siRNA targeted with G-fectin (Genolution Pharmaceuticals, Seoul, Korea) according to manufacturer's instruction. siRNA for control (5′-CCUACGCCACCAAUUUCGU-3′) and BIS (5′-AAGGUUCAGACCAUCUUGGAA-3′) were purchased from Bioneer (Daejeon, Korea). Si-14-3-3*γ* (5′-GCGAGCAACUGGUGCAGAA-3′) was purchased from Genolution Pharmaceuticals (Seoul, Korea).

### 2.6. Gelatin Zymography

Gelatin zymography was performed using supernatants from sphere media cultivated in sphere-forming media on ultralow attachment plate as previously described [33]. Briefly, conditioned media was separated by 10% SDS-PAGE containing 0.2% gelatin. The gel was renatured with 2.5% Triton X-100 buffer for 1 h and incubated with developing solution (50 mM Tris, pH 7.5, and 10 mM CaCl_2_) for 18–20 h at 37°C. The gel was then stained with 0.5% Coomassie Brilliant Blue (Sigma-Aldrich) in 30% methanol and 10% glacial acetic acid and destained with the same solution without dye.

### 2.7. Statistics

The Student* t*-test was used to compare the differences between two groups. Each experiment was repeated at least three times and *p* values of <0.05 were considered as statistically significant.

## 3. Results

### 3.1. Effects of Single or Cotreatment with PPAR*γ* Ligand and GW9662 on Glioblastoma Spheres

**Figure 1:**
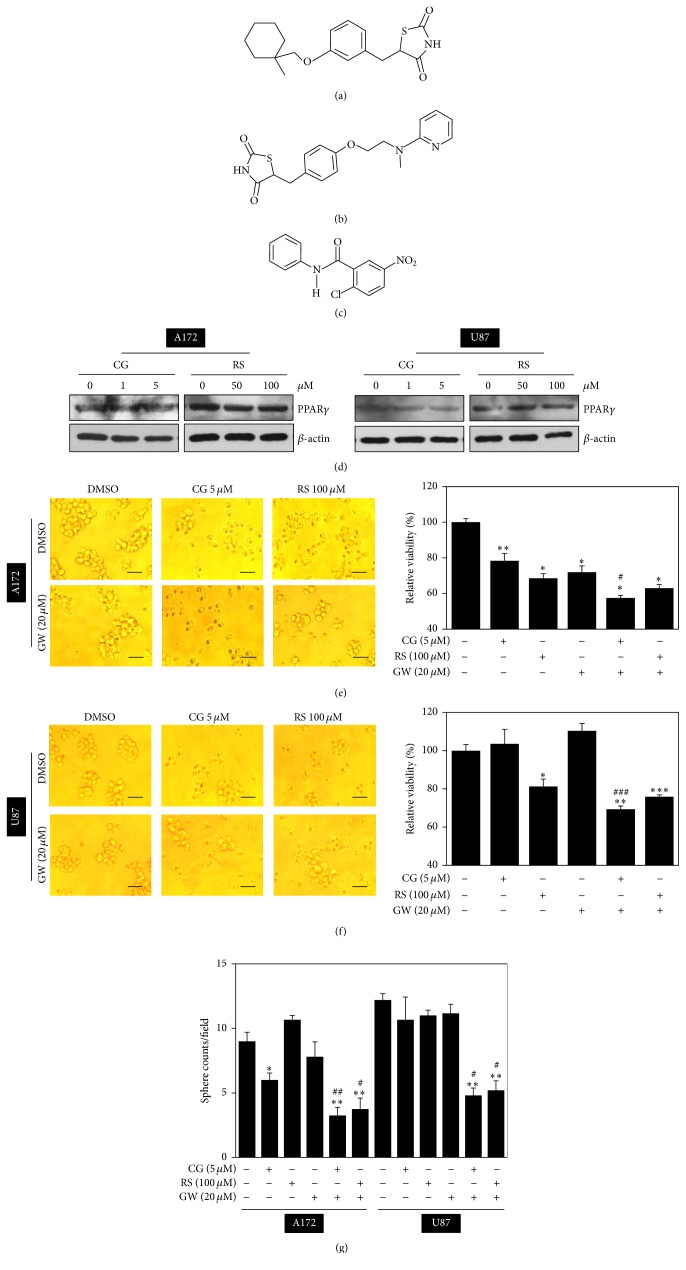
Structures of chemicals. (a) Ciglitazone (CG), C_18_H_19_N_3_O_3_S, and MW 357.428. (b) Rosiglitazone (RS), C_18_H_23_NO_3_S, and MW 333.44. (c) GW9662 (GW), C_13_H_9_ClN_2_O_3_, and MW 276.7. (d) Effects of CG and RS on protein levels of PPAR*γ*. (e) and (f) combined treatments with PPAR*γ* ligand and GW9662 decreased sphere-forming activity and viability in A172 and U87 spheres. After treatment with PPAR*γ* ligand (CG or RS) with or without GW at the indicated concentrations for 48 h, spheres were photographed under an inverted microscope, cellular viability was measured using an assay kit and (g) spheres per field were counted as described in Materials and Methods. Scale bars: 100 *μ*m. ^*∗*^*p* < 0.05, ^*∗∗*^*p* < 0.01, and ^*∗∗∗*^*p* < 0.005 versus control group without treatment. ^#^*p* < 0.05, ^##^*p* < 0.01, and ^###^*p* < 0.005 versus only GW treated group.

We verified that glioblastoma A172 and U87 cells express PPAR*γ* and single treatment with ciglitazone (CG) or rosiglitazone (RS) at a concentration tested in this study did not affect protein levels of PPAR*γ* as determined by western blotting (Figure 1(d)). To test our hypothesis that combined treatment with PPAR*γ* ligands and the PPAR*γ* inhibitor GW9662 (GW) yields a more favorable antiproliferative effect in glioblastoma spheres compared to single-agent treatments, we first examined its effects on cellular viability. To address this question, we utilized an in vitro sphere culture system [16] in which glioblastoma cells exhibited increased levels of SOX2, a representative stemness-related marker [34]. After treatment of a monolayer or spheres cultivated for 48 h with these agents, a WST-1 assay was performed as described in Materials and Methods. Combined treatment with PPAR*γ* ligands and GW9662 (Figure 1(a)) for 48 h resulted in a moderate cooperative antiproliferative effect on A172 and U87 spheres compared with single treatment (Figures 1(e) and 1(f)). The morphology of monolayers was not significantly altered (data not shown), whereas sphere-forming ability was reduced upon combined treatment with PPAR*γ* ligand and GW9662 compared with single-agent treatment (Figure 1(g)).

### 3.2. Cotreatment with PPAR*γ* Ligand and GW9662 Decreased BIS, 14-3-3 Gamma, and SOX2 Together with Reduced Phosphorylation of STAT3 and AKT

**Figure 2:**
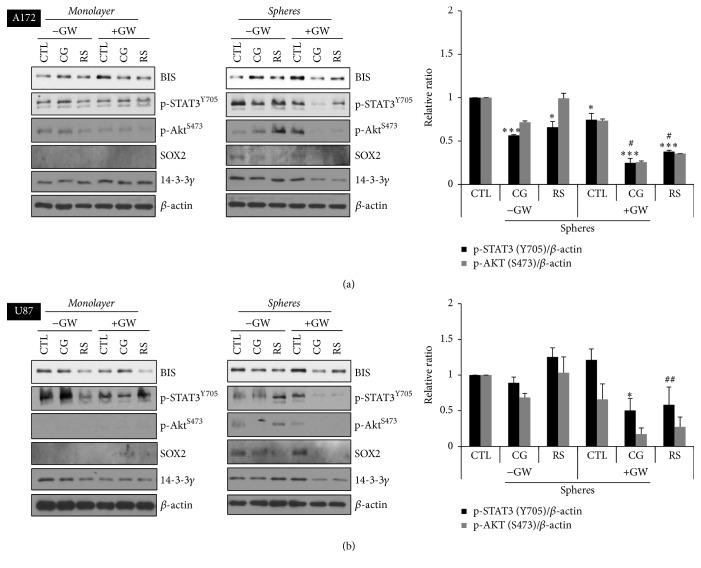
Combined treatment with PPAR*γ* ligand and GW9662 downregulated survival-related signaling molecules and stemness-related marker protein SOX2. After treatment with PPAR*γ* ligands (CG, 10 *μ*M ciglitazone; RS, 100 *μ*M rosiglitazone) with or without 20 *μ*M GW9662 (GW) for 48 h, monolayer cells or spheres of A172 (a) or U87 (b) were analyzed via western blotting with specific antibodies and the intensity of each protein per *β*-actin was measured using ImageJ software (right panels) as described in Materials and Methods. ^*∗*^*p* < 0.05 and ^*∗∗∗*^*p* < 0.005 versus control group without treatment. ^#^*p* < 0.05 and ^##^*p* < 0.01, versus only GW treated group.

Since STAT3 and AKT are well-known regulators of proliferation and stemness, we analyzed their protein levels in a monolayer and in spheres after single or combinatorial treatment via western blotting. Combined treatment decreased SOX2 protein levels in spheres compared with a monolayer (Figures 2(a) and 2(b)). Similarly, both BIS and 14-3-3 gamma were moderately reduced upon cotreatment with PPAR*γ* ligand and GW9662. Moreover, the combined treatment partially inhibited phosphorylation of both STAT and AKT in A172 and U87 spheres, indicating that the inhibitory effect on proliferation is mediated through the cellular survival pathway.

### 3.3. Effect of BIS or 14-3-3*γ* Knockdown on Cellular Viability, MMP2, and SOX2

**Figure 3:**
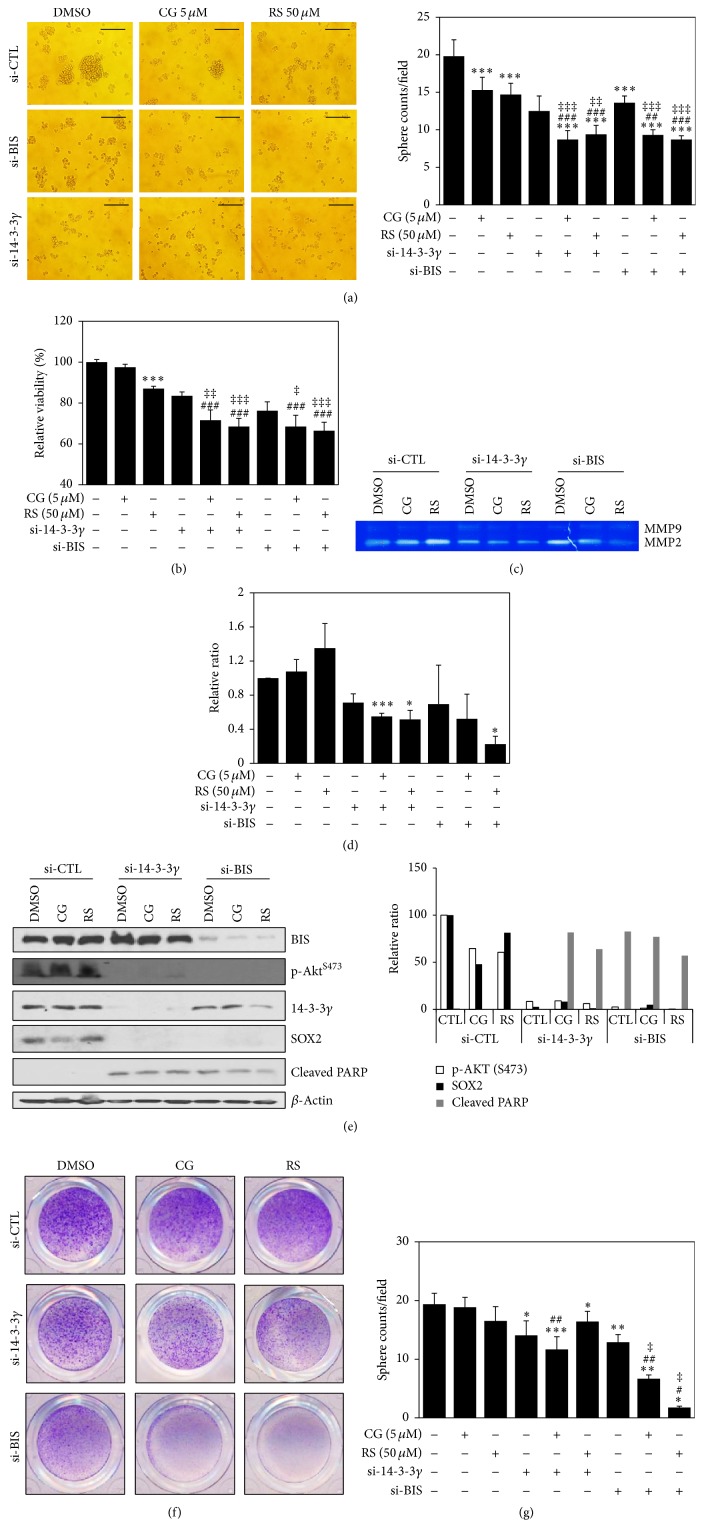
Downregulation using siRNA-enhanced PPAR*γ* ligand-induced cell death in A172 spheres together with inhibition of matrix metalloprotease 2 (MMP2) activity. After transfection with 100 nM siRNA (si-CTL, si-14-3-3*γ*, or si-BIS), cells were transferred to sphere-forming conditions with or without PPAR*γ* ligands (CG, 5 *μ*M ciglitazone; RS, 50 *μ*M rosiglitazone) for 48 h. Morphological spheres (a) were photographed under an inverted microscope and spheres per field (right panel) were counted, followed by viability (b), zymography (c and d), and western blotting (e) and spheres per field were counted (g) after crystal violet staining (f) as described in Materials and Methods. ^*∗*^*p* < 0.05, ^*∗∗*^*p* < 0.01, and ^*∗∗∗*^*p* < 0.005 versus control group without treatment. ^#^*p* < 0.05 and ^##^*p* < 0.01 versus only CG- or RS-treated group. ^‡^*p* < 0.05 versus only si-BIS or si-14-3-3*γ* treated group. ^###^*p* < 0.005 versus only CG- or RS-treated group. ^‡‡^*p* < 0.01 and ^‡‡‡^*p* < 0.005 versus only si-BIS or si-14-3-3*γ* treated group.

To address whether downregulation of BIS and 14-3-3 gamma by combined treatment with PPAR*γ* ligands and GW9662 sensitizes glioblastoma cells towards inhibition of proliferation, we performed knockdown experiments silencing BIS and 14-3-3 gamma. Cells were treated with a PPAR*γ* ligand agent for 48 h and a WST-1 assay was performed. Single treatment with si-BIS or si-14-3-3*γ* siRNAs induced morphological changes in spheres compared with the si-CTL-treated control group (Figure 3(a)). Furthermore, cotreatment with siRNA and PPAR*γ* ligand significantly enhanced the inhibitory effect of WST activity (Figure 3(b)). Since cancer stem cells display a high potential for epithelial mesenchymal transition (EMT), which is subsequently linked to an invasive phenotype, we examined matrix metalloprotease (MMP) activity as a critical agent for EMT [36, 37]. Data from zymography showed that combined treatment with 14-3-3 gamma depletion and CG or RS significantly inhibited MMP2 activity, while combination treatment with BIS depletion and RS did not (Figures 3(c) and 3(d)). To examine whether these combination treatments might affect AKT, stemness, and apoptosis, we performed western blotting assays using specific antibodies. Single treatment with si-14-3-*γ* or si-BIS decreased phosphorylation of AKT and SOX2 levels without enhancing poly(ADP-ribose) polymerase (PARP) cleavage (Figure 3(e) and right panel). Finally, we examined whether 14-3-3 gamma or BIS depletion using siRNA affects sphere-forming activity. Single treatment with si-14-3-3*γ* or si-BIS decreased sphere-forming activity and cotreatment with CG or RS moderately inhibited sphere-forming activity (Figures 3(f) and 3(g)).

## 4. Discussion

**Figure 4:**
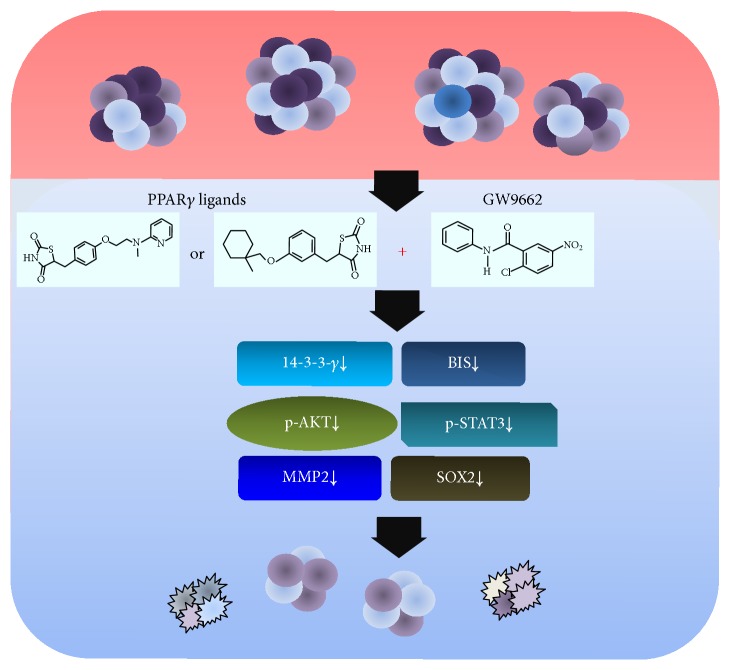
A scheme of the combined effects of PPAR*γ* ligand and GW9662 on human glioblastoma cells under sphere-forming conditions. The schematic diagram demonstrates that combination treatment with PPAR*γ* ligand (ciglitazone or rosiglitazone) and GW9662 downregulates BIS, 14-3-3 gamma, and activation of survival signaling molecules (p-AKT, p-STAT3) together with suppression of SOX2 and MMP2 activity, inhibiting stem cell-like properties and leading to apoptosis.

In this study, we demonstrated that a combined treatment with PPAR*γ* ligand and its inhibitor GW9662 downregulated BIS and 14-3-3 gamma expression and decreased phosphorylation of STAT3 and AKT, leading to inhibition of GSC-like spheres and SOX2 expression (Figure 4). Combined administration of PPAR*γ* ligand and its antagonist targeted 14-3-3 gamma and BIS, inhibiting stem cell-like properties in GSC-like spheres expressing SOX2. Our findings provide additional evidence that BIS may be a potential target in glioblastoma [38].

Emerging studies are focusing on combination therapy, such as chemo- and radiotherapy, to manage resistance from anticancer drug administration. We also found that A172 and U87 glioblastoma cells express PPAR*γ* using western blotting analysis. Hence, its ligands have been suggested as potential targets for glioblastoma stem cells [35]. Our findings provide a BIS- and 14-3-3 gamma-mediated inhibitory mechanism for the GSC-like sphere system suggesting the potential of combination therapy targeting GSCs via the manipulation of the BIS and/or 14-3-3 gamma/SOX2 axis in which STAT3 and AKT function upstream. It should be noted that 14-3-3*γ* or BIS silencing itself resulted in the considerable decrease in SOX2 levels as well as increase in PARP cleavage without synergistic and additive effect of being combined with CG or RS.

Since it has been reported that invasion is the hallmark of malignant glioblastoma and MMP [37, 39] is considered as an important player and a potential target, zymography was performed using supernatant of spheres after respective siRNA transfection to A172 cells. Single treatment with PPAR*γ* ligands did not significantly inhibit MMP2 activity but cotreatment with si-BIS or si-14-3-3*γ* inhibited MMP2 activity suggesting that these molecules might play a role in regulating MMP2 activity. It was consistent with findings from previous studies that BIS inhibits MMP2 activity [33, 40]. Meanwhile, Kim et al. [41] reported that CG increased the expression of MMP-2, and GW9662 attenuated the CG-induced PPAR*γ* activation but it did not affect the pro-MMP2 activation in a fibrosarcoma cell line HT1080. Although there is difference in cellular context, significant inhibition of MMP2 activity by CG or RG single treatment in this study was not observed.

We tested whether si-14-3-3 beta and gamma affect sphere-forming activity and found that single treatment with si-14-3-3 gamma led to moderately reduced sphere-forming activity compared to si-14-3-3 beta (data not shown), meaning that 14-3-3 gamma may play a significant role in glioblastoma stemness. Each isoform appears to differentially modulate cellular progression. 14-3-3 beta negatively regulates senescence in glioblastoma cells through the ERK pathway [42] and 14-3-3 gamma controls stem cell-like properties, as described in this study. We observed that 14-3-3 gamma or BIS deletion downregulated the SOX2 expression level without enhancing PARP cleavage, suggesting that combined treatment with PPAR ligand and its inhibitor may modulate the stemness-related pathway through AKT and STAT3 rather than the apoptosis-related pathway. Frasson et al. reported that the PI3K/AKT pathway is closely related to the stemness of medulloblastoma cancer stem cells [43], supporting our findings of combined administration in the downregulation of SOX2 in glioblastoma cells. We previously reported that BIS depletion degrades STAT3 protein leading to inhibition of sphere-forming activity [33].

As an important survival signaling kinase, AKT phosphorylates BAD at Ser136 promoting binding of BAD to 14-3-3 proteins, preventing an association between BAD and BCL-2 and BCL-xL [44]. 14-3-3 sequesters BAD in the cytoplasm under physiologically normal conditions. When cells are stressed, BAD is phosphorylated and this phosphorylated BAD (p-BAD) translocates from the cytoplasm into mitochondria leading to apoptosis. Although BIS forms an immunoreactive complex with 14-3-3 zeta [45], suggesting that BIS may affect BAD directly or indirectly through 14-3-3, we did not find that BIS or 14-3-3 gamma depletion enhanced PPAR*γ* ligand-induced PARP cleavage. Rather, single treatment significantly downregulated SOX2 expression levels. Hence, they may act directly or indirectly upon SOX2. The underlying mechanism by which BIS or 14-3-3 beta regulates SOX2 expression requires further investigation.

## 5. Conclusions

Combination treatment of GCSs with PPAR*γ* ligand and its inhibitor GW9662 inhibited stem-like properties via downregulation of BIS and 14-3-3 gamma levels together with decreased SOX2 and MMP2 activity without enhancing PARP cleavage.
